# Targeting NOTCH3 to eradicate dormant and therapy-resistant multiple myeloma cells

**DOI:** 10.1186/s13046-025-03630-1

**Published:** 2026-01-05

**Authors:** Hayley M. Sabol, Bethany C. Paxton, Aric Anloague, Japneet Kaur, Mattie R. Nester, Sharmin Khan, James Smith, Peter I. Croucher, Michelle M. McDonald, Corey O. Montgomery, Jeffrey B. Stambough, C. Lowry Barnes, Elena Ambrogini, Frank H. Ebetino, Carolina Schinke, Cody Ashby, Jesús Delgado-Calle

**Affiliations:** 1 Department of Physiology and Cell Biology, University of Arkansas for Medical Sciences, 4301 W. Markham St., 5, Little Rock, 7220 AR USA; 2 Winthrop P. Rockefeller Cancer Institute, University of Arkansas for Medical Sciences, Little Rock, AR USA; 3 Garvan Institute of Medical Research, Sydney, Australia; 4 Faculty of Medicine and Health, The University of Sydney, Sydney, Australia; 5 Department of Orthopedic Surgery, University of Arkansas for Medical Sciences, Little Rock, AR USA; 6 Division of Endocrinology and Metabolism, University of Arkansas for Medical Sciences, Little Rock, AR USA; 7 Center for Musculoskeletal Disease Research, University of Arkansas for Medical Sciences, Little Rock, AR USA; 8 Central Arkansas Veterans Healthcare System, Little Rock, AR USA; 9 Department of Chemistry, University of Rochester, Rochester, NY USA; 10 Biovinc LLC, Pasadena, CA USA; 11 Myeloma Center, University of Arkansas for Medical Sciences, Little Rock, AR USA; 12 Department of Biomedical Informatics, University of Arkansas for Medical Sciences, Little Rock, AR USA

**Keywords:** Multiple myeloma, Notch, Dormancy, Bortezomib, Resistance

## Abstract

**Background:**

Despite significant therapeutic advances, multiple myeloma (MM) remains incurable in most patients due to frequent tumor relapse. A major contributor to relapse is clonal heterogeneity, where subclones exhibit distinct mechanisms of therapy resistance, along with the presence of drug-resistant dormant cells. Eliminating these distinct populations, which often coexist in the tumor niche, is clinically challenging. Identifying survival mechanisms shared by drug-resistant proliferating and dormant cells holds potential for the simultaneous elimination of different tumor-repopulating clones.

**Methods:**

To identify shared mechanisms of therapeutic resistance, we analyzed clinical databases and drug-resistant myeloma cell lines. We employed pharmacologic approaches to target common candidates identified in our analysis and assessed their impact on tumor progression and survival in preclinical mouse models containing both therapy-resistant and dormant cells.

**Results:**

We identified upregulation of several components of the Notch signaling pathway in both dormant and drug-resistant MM cells, which correlated with poor clinical outcomes in newly diagnosed MM patients. Selective blockade of NOTCH3 with a neutralizing antibody or pan-Notch inhibition with a bone-targeted inhibitor reduced tumor burden and eliminated coexisting dormant and bortezomib-resistant cells in clinically relevant models of MM disease.

**Conclusions:**

Our findings reveal that NOTCH3-dependent survival programs represent a shared vulnerability in both cells refractory to therapy and dormant cells. These programs can be exploited to overcome the diverse mechanisms by which cancer cells evade therapy, potentially preventing disease relapse and extending remission in patients with MM.

**Supplementary Information:**

The online version contains supplementary material available at 10.1186/s13046-025-03630-1.

## Background

Multiple myeloma (MM) is a hematologic cancer characterized by the clonal proliferation of malignant plasma cells within the bone marrow [1]. MM remains incurable, as most patients experience relapse after initial therapy, even those who achieve complete remission and have no detectable minimal residual disease (MRD) by molecular methods [2]. Although MM patients now have access to novel treatment options in the relapse setting, their efficacy and duration remain limited, with the disease often progressing to refractory stages. Therefore, effective management of relapsed/refractory disease remains crucial to improving clinical outcomes in MM patients.

Clonal heterogeneity and the emergence of treatment-resistant clones are key features of disease relapse in MM [3]. Current upfront regimes combine proteasome inhibitors, dexamethasone, immunomodulatory agents, and CD38 monoclonal antibodies, followed by high-dose chemotherapy and autologous stem cell transplantation for eligible patients. The development of therapy resistance during or after such intensive regimens highlights a complex and dynamic process driven by both cell-intrinsic properties, some of which may exist before therapy, and extrinsic mechanisms shaped by microenvironmental signals during and after therapy [4–6]. Additionally, cancer cell dormancy also contributes to therapeutic resistance. Reservoirs of cell cycle-arrested, drug-resistant, dormant cells with tumor-repopulating potential have been identified in MM [7–9]. Moreover, subclones exhibiting distinct therapy resistance and dormancy can coexist within the same MM patient [9], posing a significant clinical challenge.

One potential strategy to overcome drug-resistant clonal diversity and dormancy is to identify shared vulnerabilities across different therapy-resistant cell populations for simultaneous eradication. Notch signals have been shown to contribute to secondary resistance mediated by cells of the tumor niche [10–15]. In this study, we demonstrate that NOTCH3 is a common vulnerability in both dormant and MM cells refractory to the proteasome inhibitor Bortezomib (BOR), a cornerstone therapy for MM. Further, we show that selective blockade of NOTCH3 or bone-targeted pan-Notch inhibition effectively eliminates resistant and dormant cells in clinically relevant models of MM. The results of this study indicate that safe Notch-directed therapies hold the potential to overcome disease relapse and extend remission in MM patients.

## Methods

### Cells and reagents

Murine 5TGM1 MM cells (RRID: CVCL_VI66) and human OPM2 MM (RRID: CVCL_1625) cells were provided by Dr. Oyajobi (University of Texas at San Antonio, TX, USA) and Dr. Roodman (Indiana University). 5TGM1 parental and bortezomib-resistant cells (BOR-R) were provided by Dr. Xing (University of Rochester, NY, USA) [16]. U266 and RPMI-8226 parental and bortezomib-resistant cells (BOR-R) were provided by Dr. Steven Grant (Virginia Commonwealth University, VA, USA) [17]. MM cell lines were cultured as previously described and authenticated for proper morphology, surface markers, and paraprotein production [10, 18]. RPMI 1640 media, fetal bovine serum, Normocin, Plasmocin, antibiotics (penicillin/streptomycin), TriZol, and 1’-dioctadecyl-3,3,3’,3’-tetramethylindodicarbocyanine (DiD) cell trackers (Cat#V-22887) were purchased from Invitrogen Life Technologies (Grand Island, NY, USA). Puromycin (Cat#58-58-2) and Blasticidin (Cat# ant-bl) were purchased from InvivoGen (San Diego, CA, USA). Melphalan (Cat# M2011) was purchased from Sigma-Aldrich (St. Louis, MO, USA). The neutralizing antibodies against murine and human NOTCH3 (NOTCH3-ab) were provided by AVEO Oncology, an LG Chem company (Boston, MA, USA).

### Generation, pharmacokinetics, and pharmacodynamics of BT-GSI

The bone-targeted γ-secretase inhibitor (GSI) (BT-GSI) was generated as described before [18]. BT-GSI is a chemical conjugate that incorporates (i) a modified, less active bisphosphonate moiety with high bone affinity designed to direct the conjugate to the skeleton, (ii) a pH-sensitive labile moiety that covalently links the “cargo” to the bisphosphonate, and (iii) the small molecule cargo GSI-XII, which is known to inhibit Notch signaling by preventing the cleavage of the intracellular domain of Notch receptors by blocking the activity of the γ-secretase complex. To investigate the pharmacological properties of BT-GSI, we conducted studies in mice treated with (1) equimolar doses of BT-GSI (10 mg/kg, 3x/week) or unconjugated GSI (5 mg/kg, 3x/week) for 4 weeks or with (2) a single dose of BT-GSI. Serum and bone tissues were collected at 1 h, 2 h, 4 h, 8 h, and 24 h post-treatment. Free GSI concentrations were quantified using HPLC-MS/MS by the Clinical Pharmacology Analytical Core (Indiana University School of Medicine, IN, USA). After 4 weeks of treatment, mice receiving BT-GSI had a 4-fold higher concentration of free GSI in the bone and an 8-fold lower concentration of GSI in serum compared to those treated with unconjugated GSI (Fig. S1A). Serum levels of free GSI decreased within 2 h and became undetectable by 24 h, whereas bone concentrations remained stable after 8 h and were detectable even at 24 h (Fig. S1B). Moreover, the concentration of free GSI in bone correlated with the percent inhibition of the Notch target genes, *Hey1*, *Hey2*, and *Hes5* (Fig. S1C). These results demonstrate that our pharmacological strategy directs the GSI to the bone, achieving local and sustained concentrations of free GSI capable of decreasing Notch signaling.

### Animal studies

MM cells were labeled with the membrane dye DiD. DiD labels the cell membrane, and its mean fluorescence intensity decreases as MM cells divide, with the label progressively lost as it is distributed among daughter cells, ultimately resulting in a negative signal [7, 8]. 8-week-old C57BL/KaLwRijHsd female and male mice [19–21] were injected intravenously with 10^6^ DiD^+^GFP^+^ 5TGM1 MM cells. After 1 week, mice were randomized by tumor burden and treated with BT-GSI (2.5 mg/kg; 3x/week, i.p.) for 8 or 2 weeks. Another subset of double-labeled 5TGM1-injected C57BL/KaLwRijHsd mice were randomized based on tumor burden 2 weeks after cell inoculation to [1] vehicle, BT-GSI, a neutralizing antibody against sclerostin (Scl-ab; 100 mg/kg; 1x/week, i.p.), or BT-GSI + Scl-ab (combo), and sacrificed after 1 or 2 weeks. 8-week-old NSG female and male mice were injected intravenously with 2 × 10^6^ OPM2 mCherry^+^-DiD^+^ MM cells. After 2 weeks, mice were randomized to vehicle or BT-GSI groups based on tumor burden and sacrificed after 1 week. 7-week-old immunocompetent C57BL/KaLwRijHsd female and male mice were pre-treated with veh or Scl-ab for 2 weeks, then injected intravenously with 10^6^ DiD^+^GFP^+^ 5TGM1 MM cells, and treated with veh or Scl-ab for 3 additional weeks. 8-week-old immunocompetent C57BL/KaLwRijHsd female and male mice were injected intravenously with 10^6^ DiD^+^GFP^+^ 5TGM1 parental or bortezomib-resistant (BOR-R) cells. After 1 week, mice were randomized to treatment groups and received vehicle, BT-GSI, NOTCH3-antibody (NOTCH3-ab; AVEO Oncology, 20 mg/kg 3x/wk, i.p.), or bortezomib (BOR; 0.6 mg/kg 3x/wk, i.p.) for 3 weeks. To assess survival, the health of mice was monitored daily, and mice were euthanized at the first signs of back leg paralysis. Group sizes were selected based on prior similar experiments published by our group [10, 18, 22].

### *Ex vivo* organ cultures

Ex vivo MM-human bone organ cultures were established using human cancellous bone fragments of similar size, obtained from femoral heads discarded after hip arthroplasty, as described previously [10, 23]. Bone fragments and 200,000 OPM2 mCherry^+^DiD^+^ MM cells were incubated for 24 h. After 24 h, bones with infiltrated MM cells were moved to a new plate with fresh media and treated with vehicle, melphalan (1µM; treated 1x/wk), or BT-GSI (15µM; treated 2x/wk). MM cells were isolated from the bone and analyzed by flow cytometry. Murine tibiae or calvarial disks and 200,000 5TGM1 (parental or BOR-R) cells or human bone fragments and 200,000 RPMI-8226 or U266 (parental or BOR-R) were incubated for 24 h. After 24 h, bones with infiltrated MM cells were transferred to a new plate with fresh media and treated with vehicle, BOR (5nM; treated 2x/wk), VRd (BOR 2nM, Lenalidomide 1µM, Dexamethasone 10nM; treated 2x/wk), BT-GSI (15µM; treated 2x/wk), NOTCH3-ab (20 µg/mL; treated 2x/wk), BOR + BT-GSI, BOR + NOTCH3-ab, VRd + BT-GSI, or VRd + NOTCH3-ab. Treatments were refreshed every 3 days, and conditioned media was collected after 11 days.

### Flow cytometry

The percentage of GFP^+^/mCherry^+^ and DiD^Hi^ MM cells in the bone marrow was assessed using flow cytometry (LSR Fortessa; BD Biosciences, San Jose, CA, USA). The bone marrow from 3 long bones was flushed out, red blood cells were lysed, and the remaining cells were stained with PerCP 5.5 Annexin V (BD Biosciences, San Jose, CA, USA; Cat# 561431), DAPI, or Axl (R&D Systems, Minneapolis, MN, USA; Cat# FAV8541P). For cell cycle analysis, samples were GFP^+^ sorted, fixed, and stained with DAPI and Ki-67 (Thermo Fisher; Waltham, MA, USA; Cat# 61-5698-80). The samples were analyzed using FlowJo (Ashland, OR, USA).

### Apoptosis and viability assays

5TGM1, U266, and RPMI-8226 parental and BOR-R cells were treated with veh, BOR (3nM), GSI (15µM), and/or NOTCH3-ab (20 µg/mL) for 48 h. 5TGM1 cells were stained with Annexin and DAPI and analyzed in a BD FACS Calibur (UAMS Core Facility for Flow cytometry) within 1 h. At least 10,000 cells were used for each group. The samples were analyzed using FlowJo (Ashland, OR, USA). U266 and RPMI-8226 cells were collected and counted using trypan blue.

### RT-qPCR

Total RNA was isolated from bone tissues using Trizol and converted to cDNA (Invitrogen Life Technologies), following the manufacturer’s directions. Gene expression was quantified by quantitative real-time PCR (qPCR) using TaqMan assays from Applied Biosystems (Foster City, CA, USA), according to the manufacturer’s instructions. Gene expression levels were calculated using the comparative threshold (CT) method and were normalized to the housekeeping gene GAPDH.

### microCT

microCT imaging was performed in live mice using a vivaCT 80 (Scanco Medical AG, Switzerland). Bones were aligned to the proximal axis, and analysis of the cancellous bone was performed in the tibia, as shown before [10, 18].

### Enzyme-linked immunoassays (ELISA)

The levels of the tumor biomarker IgG2B (Invitrogen; Cat#88-50430-88), a paraprotein produced by 5TGM1 cells, or human lambda light chain (Bethyl Laboratories, Montgomery, TX, USA; Cat#E88-116), were used to determine tumor growth/burden in vivo (serum) or ex vivo (conditioned media).

### Western blot

Cell lysates (50 µg) were boiled in the presence of SDS sample buffer (NuPAGE LDS sample buffer; Invitrogen) for 10 min and then subjected to electrophoresis on 10% SDS-PAGE (Bio-Rad Laboratories). Proteins were transferred to PVDF membranes using a semidry blotter (Bio-Rad) and incubated in a blocking solution (5% nonfat dry milk in TBS containing 0.1% Tween-20) for 1 h to reduce nonspecific binding. Immunoblots were performed using anti-GAPDH (Cat#2118S, RRID: AB_561053, Cell Signaling Technologies, Danvers, MA, USA), NOTCH3 (ab23426, RRID: AB_776841, Abcam, Cambridge, MA, USA), and HES1 (ab71559, RRID: AB_1209570, Abcam, Cambridge, MA, USA) antibodies (1:1000) overnight at 4 °C followed by goat anti-rabbit secondary antibodies, conjugated to horseradish peroxidase (1:2000) in 5% milk (Santa Cruz Biotechnology). Western blots were developed using an enhanced chemiluminescence detection assay following the manufacturer’s directions (BioRad). Protein bands were quantified using ImageJ.

### Patient survival analysis and dormancy signature

Enrichment scores were calculated using the Gene Set Variation Analysis (GSVA) package in R to assess gene sets associated with dormancy [7] and proliferation [24] (see Supplementary Table 1 with gene sets). Salmon gene count data were imported into and normalized using the R package DESeq2. To determine patients with high versus low dormancy signatures, human homologs of dormant mouse genes were identified using Ensembl version 112, with the ortholog_one2one homology type. The resulting genes were filtered to ensure uniqueness and presence in the CoMMpass data, yielding two distinct gene sets: one comprising overexpressed genes (up_genes) and another comprising downregulated genes (down_genes) in dormant cells. GSVA enrichment scores were then calculated for these gene sets using default values. To classify samples, a sample was considered to have high dormancy if its enrichment score was positive and greate than 0 for the up_genes list and negative for the down_genes list. Conversely, samples with enrichment scores less than 0 in the up_genes list and greater than 0 in the down_genes list were classified as low dormancy. For proliferation, samples were classified as having high proliferation if their enrichment score was greater than 0; otherwise, they were classified as having low proliferation. We segregated patients based on the gene expression of the components of the γ-secretase complex and performed survival analysis as described previously [10, 25].

### Isolation and culture of primary CD138^+^cells from MM patients

Cryopreserved CD138 + cells from patients (median age 66.9, 60% female, 80% caucasian) who reached partial responses (MRD positive) after first therapy (including BOR, dexamethasone, daratumumab, melphalan, carfilzomib, or lenalidomide) were obtained from the Tissue Biorepository and Procurement Service. CD138^+^ cell isolation was performed using immunomagnetic bead selection with a monoclonal mouse anti–human CD138 antibody and the AutoMACS automated separation system (Miltenyi-Biotec). Purity was confirmed by 2-color flow cytometry with the use of CD138+/CD45 − criteria (Becton Dickinson), immunocytochemistry for cytoplasmic light-chain immunoglobulin, and morphology by Wright-Giemsa staining. CD138^+^ cells were treated with veh, GSI (30µM), or human NOTCH3-ab (40 µg/mL) for 48–72 h. Apoptosis was determined via flow cytometry, as described above.

### Statistical analysis

Data acquisition and analysis were performed in a blinded fashion. Based on previous data [7, 8, 10, 18, 26], we estimated that to detect a 50% decrease in mean tumor volume/dormancy with 90% power at a significance level of α = 0.05, a sample size of *n* = 4 mice per group was necessary for T-test comparisons, and *n* = 6–8 mice per group for one-way ANOVA comparisons. Data were analyzed using GraphPad (GraphPad Software Inc., San Diego, CA, USA). The data distribution was assessed using the Shapiro-Wilk test when possible. Differences in means were analyzed using a combination of lognormal and ordinary paired t-tests, or One-Way tests, followed by pairwise multiple comparisons (Tukey/Dunnett post hoc tests), as indicated in the figure legends. Data exceeding two standard deviations from the mean were considered outliers and removed from the analysis. Values were reported as means ± SD, unless otherwise indicated in the figure legends.

## Results

### Bone-targeted Notch inhibition eradicates dormant MM cells

**Fig. 1:**
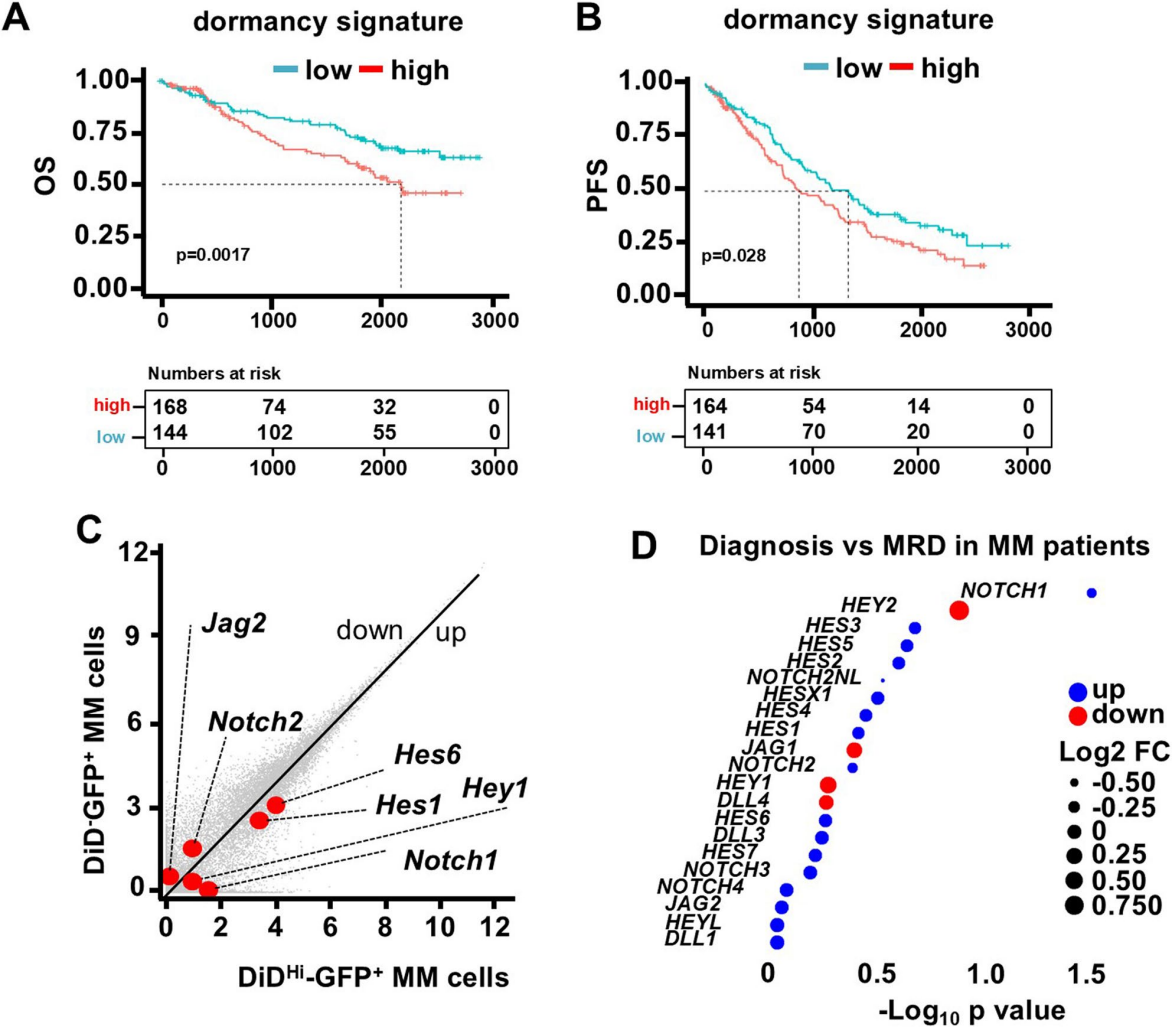
Notch target genes are upregulated in dormant MM cells and MM patients with MRD. Kaplan-Meier plot of the (**A**) overall survival (OS) and (**B**) progression-free survival (PFS) of NDMM patients with high vs. low dormancy signature expression. n=373 patients. Data were analyzed using a Log-rank (Mantel-Cox) test. **C** Volcano plot ranking genes according to their relative abundance (log2 fold change). Red dots show significant dysregulated Notch-related genes in dormant vs. proliferating MM cells isolated from immunocompetent mice bearing 5TGM1 murine tumors. **D** Gene expression of Notch components in a published dataset (Paiva et al. Blood; 2016;127(15):1896) composed of paired CD138+ plasma cell samples from MRD-positive MM patients after therapy versus at diagnosis (n=7 patients). Bubble size is proportional to the Log2 fold change. Data were analyzed using a Benjamini-Hochberg test

We first focused on MM dormant cells, a relatively new population of MM cells resistant to therapy and with tumor-repopulating capacity [8, 9]. We found that newly diagnosed MM patients with a higher expression signature of dormancy genes [7] displayed poorer overall survival and progression-free survival (Fig. 1A-B). This negative association was further accentuated in patients with concurrent upregulation of a high-proliferation gene signature [24] (Figs. S2A-B). To identify potential vulnerabilities of dormant MM cells, we next mined a previously published single-cell RNA sequencing dataset of proliferating (DiD^−^GFP^+^) and dormant (DiD^Hi^GFP^+^) murine MM cells [7] with a focus on genes of the Notch pathway, which have been linked to therapy resistance [10–12, 27]. We observed upregulation of genes related to the Notch pathway (*Notch1*, *Hes1*, *Hey1*, *Hes6*) in dormant cells (Fig. 1C). Similarly, we also observed upregulation of *NOTCH1*, along with non-significant upregulation of several other Notch components, in a gene expression dataset [28] comparing paired plasma cell samples from patients who remained MRD-positive after therapy versus at diagnosis (Fig. 1D). These results suggest that Notch signals are active and might play a role in dormant-residual resistant MM cells.

**Fig. 2:**
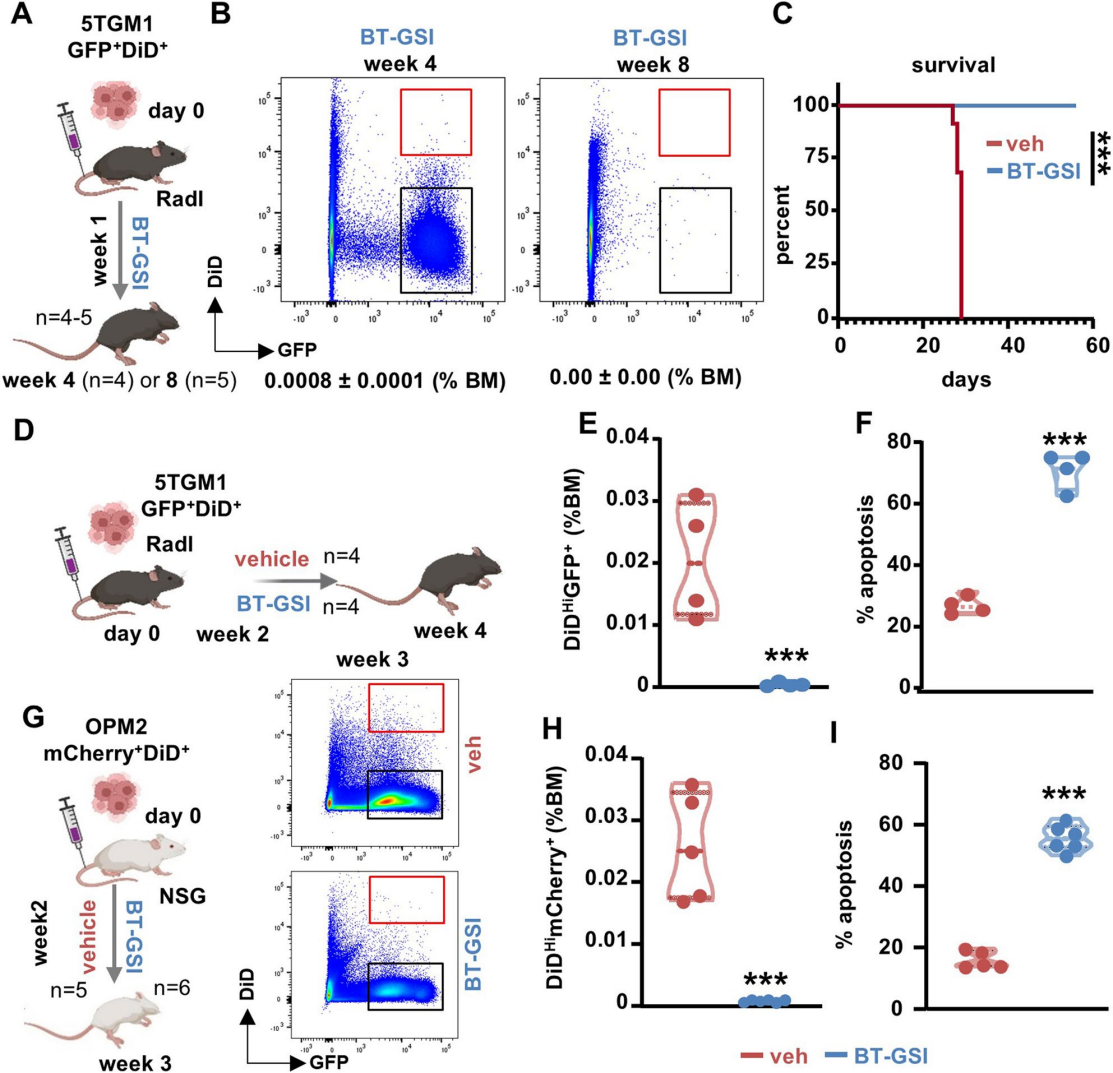
Bone-targeted Notch inhibition effectively eliminates both murine and human dormant MM cells. **A** Immunocompetent mouse model experimental design. **B** Representative flow cytometry plots of bone marrow GFP and DiD expressing 5TGM1 MM cells and (**C**) survival of mice receiving vehicle or BT-GSI at 4weeks (n=4) and 8 weeks (n=5). Red rectangles indicate the GFP+DiDHi MM dormant population, and black rectangles represent the proliferating GFP+-DiD- MM population. **D** Immunocompetent mouse model experimental design. **E-F** Percentage of dormant (GFP+DiDHi) and apoptotic dormant 5TGM1 MM cells in mice receiving vehicle or BT-GSI for 2 weeks. n=4 mice/group. **G** Immunodeficient mouse model experimental design and representative flow cytometry plots of bone marrow mCherry and DiD expressing OPM2 MM cells. **H** Percentage of mCherry+DiDHi dormant and (**I**) apoptotic dormant OPM2 MM cells from mice receiving vehicle or BT-GSI treatment for 1 week. n=5-6 mice/group. *p<0.05; **p<0.01; ***p<0.001 vs. vehicle by log-rank (Mantel-Cox) test (C) or ordinary T-test (E-F, H-I)

To study MM cell dormancy in vivo, we injected double-labeled GFP^+^DiD^+^ 5TGM1 MM cells in immunocompetent mice, a system in which only non-proliferating dormant cells retain the DiD label, which is lost with each division in proliferating cells. We identified a G0 cell-cycle arrested, Axl^+^, DiD^Hi^GFP^+^ dormant MM population in the bone marrow by flow cytometry, which persisted throughout the mouse lifespan (Figs. S3A-E). To test the impact of Notch inhibition on dormant cells, mice received treatment with a bone-targeted Notch inhibitor (BT-GSI) that suppresses Notch in bone (Fig. S1A) and CD138^+^ MM cells in the bone marrow niche [26] without causing toxicities [18]. Treatment with BT-GSI for seven weeks led to a time-dependent, complete elimination of dormant MM cells (Figs. 2A-B), a significant reduction in proliferating MM cells (Figs. S4A-B), and improved long-term survival (Fig. 2C). Treatment for 1–2 weeks with BT-GSI also reduced the proportion and number of dormant MM cells by 96–98% through the induction of apoptosis, and decreased tumor burden (Figs. 2D-F and S4C-D). Consistent with its bone-specific effects, no significant differences in the proportion of proliferating or dormant MM cells were observed in the spleen between BT-GSI and vehicle-treated groups (Figs. S4E-F). To examine the effects of BT-GSI on human dormant cells, we used a xenograft mouse model established with double-labeled (mCherry^+^DiD^+^) human OPM2 MM cells, a model for an aggressive form of MM that carries the poor-prognosis genetic mutation t(4;14) present in 15–20% of MM patients. Remarkably, 1 week of treatment with BT-GSI was also sufficient to induce apoptosis and reduce the proportion and number of human dormant MM cells by 80% in this model (Figs. 2G-I), while decreasing proliferating MM cells by 70% (Figs. S4G-I).

**Fig. 3:**
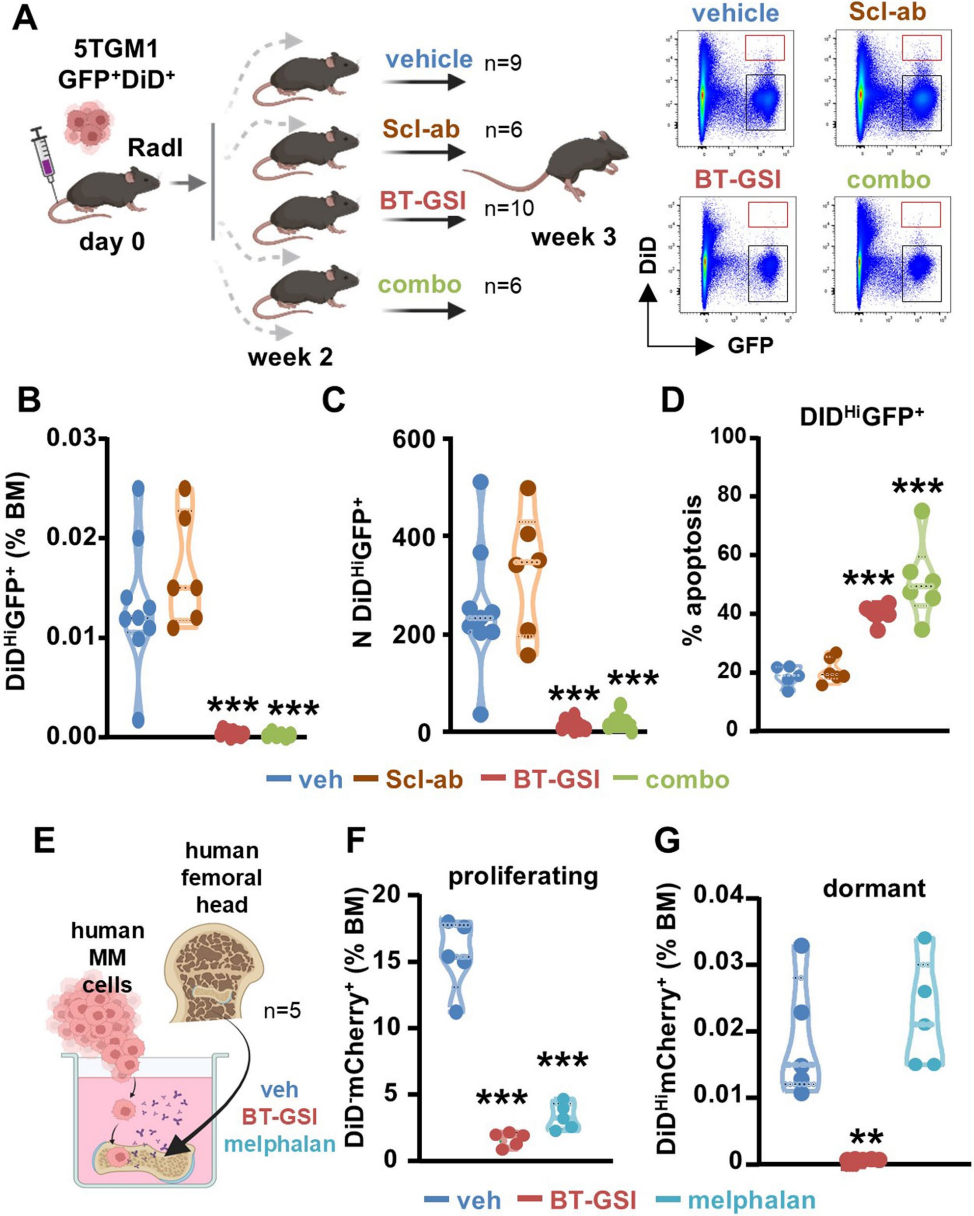
Osteoblasts do not interfere with BT-GSI's efficacy to eliminate dormant cells. **A** Immunocompetent mouse model experimental design and representative flow cytometry plots of bone marrow GFP and DiD 5TGM1 MM cells. Red rectangles indicate the GFP+DiDHi MM dormant population, and black rectangles represent the proliferating GFP+DiD- MM population. **B-C** Percentage and number of GFP+DiDHi dormant 5TGM1 MM cells in mice receiving vehicle, Scl-ab, BT-GSI, or BT-GSI+Scl-ab (combo) for 1 week. n=6-10 mice/group. **D** Prevalence of apoptotic GFP+DiDHi dormant 5TGM1 MM cells in a randomly selected subset of mice receiving vehicle, Scl-ab, BT-GSI, or combo for 1 week. n=5-6 mice/group. **E** Ex vivo bone-MM organ cultures were established with human bone fragments from donors and human OPM2 MM cells. **F-G** Percentage of proliferating (mCherry+DiD-) and dormant (mCherry+DiDHi) OPM2 MM cells in bone cultures treated with vehicle, BT-GSI, or melphalan for 11 days. n=5 bones/group. *p<0.05; **p<0.01; ***p<0.001 vs. vehicle by ordinary one-way ANOVA followed by a Tukey post hoc test

Previous studies have shown that dormant MM cells localize near endosteal osteoblasts, which may contribute to inducing and maintaining MM dormancy and provide a protective environment [8]. Thus, we investigated whether BT-GSI retains its ability to eliminate dormant cells in the presence of a niche enriched with osteoblasts (Fig. 3A). To increase the number of osteoblasts, we treated mice with Scl-ab, a potent bone anabolic agent that markedly increases osteoblasts in bone by activating the Wnt signaling pathway in cells of the osteoblast lineage [26, 29, 30]. BT-GSI, either alone or in combination with Scl-ab, similarly reduced the percentage and number of dormant MM cells through induction of apoptosis (Figs. 3B-D), decreased the proportion of proliferating GFP^+^DiD^−^ MM cells (Figs. S5A-B), but did not affect MM-induced spleen enlargement (Fig. S5C). No significant differences were observed in the proportion or number of MM cells entering dormancy (Figs. 3B-D) or the growth of proliferating MM cells (Fig. S5B) between Scl-ab and control mice, even when the mice were pre-treated with Scl-ab before tumor inoculation (Figs. S5D-J). Lastly, we compared the effects of BT-GSI and melphalan, a mainstay MM therapy that targets actively dividing cells [8, 31], on dormancy ex vivo in a human bone niche. While BT-GSI and melphalan similarly decreased tumor burden, only BT-GSI but not melphalan reduced the percentage of dormant MM cells (Figs. 3E-G). Together, these studies support the notion that Notch signals promote the survival of MM dormant cells and identify Notch as a targetable vulnerability for eliminating dormant cells.

### NOTCH3 is upregulated in bortezomib-resistant MM cells

**Fig. 4:**
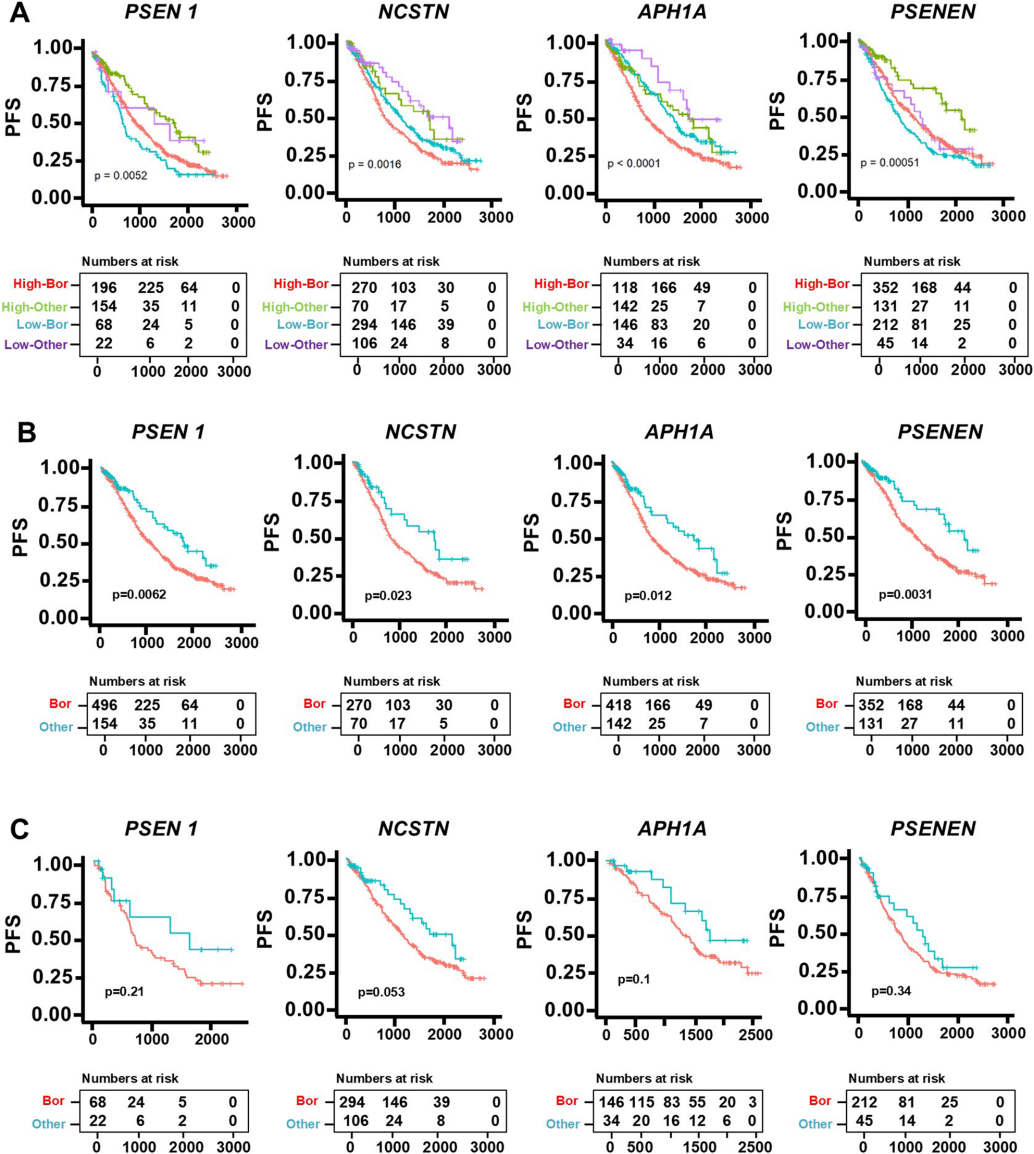
High expression of components of the γ-secretase complex correlates with worse response to BOR-based therapy. **A** Kaplan-Meier plot of the progression-free survival (PFS) of newly diagnosed MM patients with high expression of γ-secretase complex components receiving BOR-based therapies (red) or other therapies not including BOR (green), or low expression of γ-secretase complex components receiving BOR-based therapies (blue) or other therapies not including BOR (purple). **B** Kaplan-Meier plot of PFS in newly diagnosed MM patients with high expression of γ-secretase complex components or (**C**) low expression of γ-secretase complex components receiving BOR-based therapies (red) or other therapies not including BOR (blue). n=785 patients. Data were analyzed using a Log-rank (Mantel-Cox) test

We recently showed that newly diagnosed MM patients with elevated expression of Notch pathway components, particularly *NOTCH3*, exhibit poor clinical responses to bortezomib (BOR)-based therapies [10], a key treatment in MM that is often hindered by the emergence of therapeutic resistance. We also found increased expression of Notch receptors in MM patients at relapse compared to the time of diagnosis [10]. Building on these findings, we investigated the clinical impact of gene expression levels of the four components of the γ-secretase complex (*PSEN1*, *APH1A*, *NCSTN*, *PSENEN*), which are essential for Notch receptor activation and are the Notch components targeted by BT-GSI. Patients with high expression of γ-secretase complex genes who received BOR-based therapies had worse progression-free survival compared to those receiving other therapies without BOR (Figs. 4A-C).

**Fig. 5:**
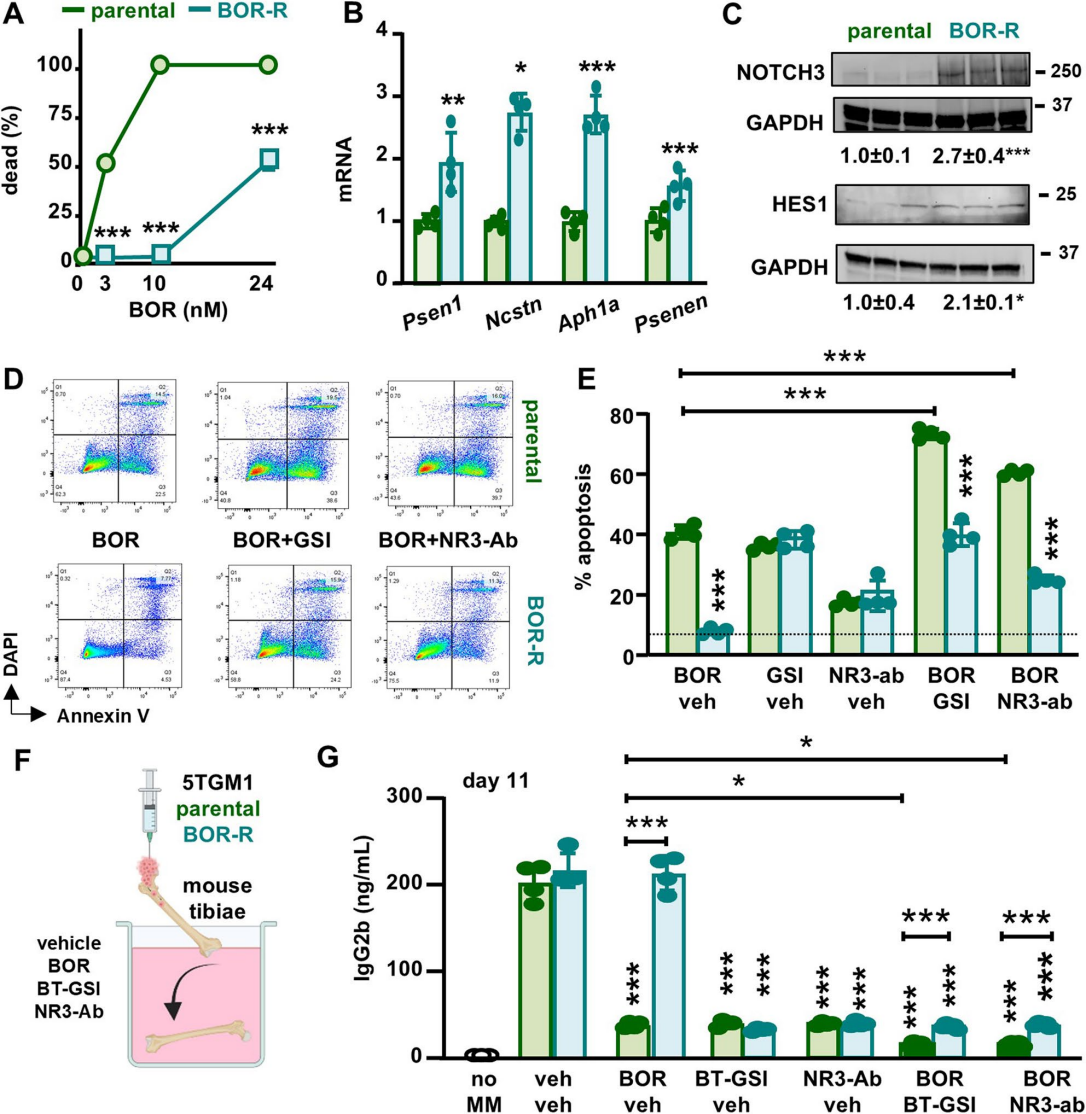
Notch signaling components are upregulated in BOR-R MM cells. **A** Dose-response of parental and bortezomib-resistant (BOR-R) 5TGM1 MM cells treated with 0-24nM BOR for 48h. **B** mRNA gene expression of γ-secretase complex components and (**C**) protein expression of NOTCH3 and the Notch target gene HES1 in 5TGM1 parental and BOR-R cells. **D** Representative flow cytometry plots and (**E**) percentage of apoptotic cells in parental and BOR-R 5TGM1 MM cells treated with veh, BOR, GSI, NOTCH3-ab (NR3-ab), BOR+GSI, or BOR+NR3-ab. n=3-4/group *p<0.05; **p<0.01; ***p<0.001 vs parental by ordinary t-test. **F** Ex vivo experimental design. **G** Levels of the tumor biomarker IgG2b in the conditioned media from ex vivo murine tibias injected with parental or BOR-R 5TGM1 MM cells treated with vehicle, NR3-ab, BOR, GSI, BOR+GSI, or BOR+NR3-ab for 11 days. n=4-5/group.*p<0.05; **p<0.01; ***p<0.001 vs. parental (veh) by ordinary T-test (A-C) or ordinary two-way ANOVA, followed by a Tukey post hoc test (E, G)

Next, we investigated the levels of Notch components in a murine 5TGM1 MM cell line with intrinsic BOR resistance (BOR-R IC_50_ = 24nM, parental IC_50_ = 3nM) (Fig. 5A), which was generated through sustained culture in the presence of increasing concentrations of BOR [16]. Compared with parental cells, BOR-R cells showed increased expression of γ-secretase complex genes, higher NOTCH3 receptor expression, and upregulation of Notch target genes (Figs. 5B-C and S6A). Similar results were observed in two human cell lines with intrinsic BOR resistance (RPMI-8226 and U266) (Figs. S6B-E). Collectively, these results suggest that MM cells resistant to BOR have upregulation of targetable Notch components, particularly NOTCH3.

### NOTCH3 inhibition induces apoptosis in bortezomib-resistant MM cells

**Fig. 6:**
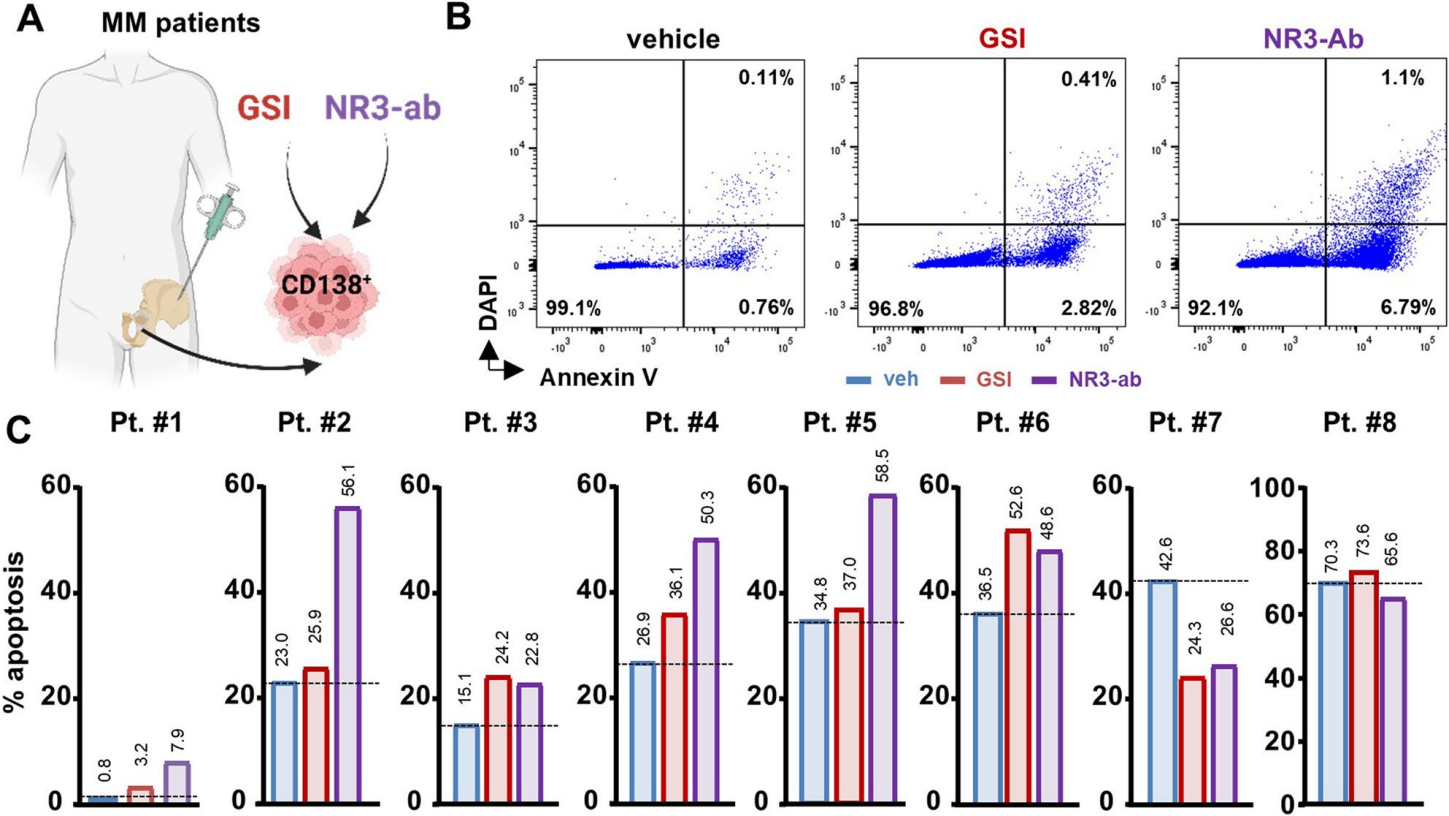
Notch inhibitors induce apoptosis in CD138+ cells from MM patients with partial response to therapy. **A** CD138+ MM cells were isolated from MM patients with partial responses to therapy. **B** Representative flow cytometry plots (patient #1) and (**C**) percentage of apoptotic cells in CD138+ cells isolated from 8 different relapsed patients treated with veh, GSI, or NOTCH3-ab (NR3-ab)

To assess the effects of Notch inhibition on BOR-R MM cells, we next treated these cells in vitro with a neutralizing antibody that selectively inhibits the activation of NOTCH3 (NOTCH3-ab) or with a GSI that inhibits all four Notch receptors. Both NOTCH3-ab and GSI induced apoptosis in both the parental and BOR-R cells when administered alone. Combination therapies of Notch inhibitors with BOR induced an additive effect in parental cells. Still, BOR did not affect BOR-R cells, either alone or in combination with Notch inhibitors (Figs. 5D-E). Similar results were obtained in BOR-R RPMI-8226 and U266 MM cells (Figs. S6F-G). We also tested the efficacy of Notch inhibitors on primary CD138^+^ cells isolated from eight MM patients with partial responses to therapy (Figs. 6A-B). GSI and NOTCH3-ab induced apoptosis in 6 out of the 8 patients, with increases ranging from 10% to 600% for GSI and 30% to 800% for NOTCH3-ab (Fig. 6C).

Next, we evaluated the efficacy of Notch inhibition in eliminating BOR-R cells using ex vivo bone organ cultures that better reproduce the complexity of the tumor niche than 2D culture systems [32]. We injected 5TGM1 parental or BOR-R cells into the explanted tibiae of mice and treated them with NOTCH3-ab or BT-GSI alone or in combination with BOR. After 11 days of culture, NOTCH3-ab and BT-GSI similarly reduced both parental and BOR-R tumors. These effects were enhanced by BOR co-administration only in bones bearing parental cells (Figs. 5F-G**)**. Similar results were observed in ex vivo organ cultures established with human RPMI-8226 and U266 parental and BOR-R cells and human bones from donors (Figs. S7A-D). Interestingly, 5TGM1 BOR-R cells responded to a triple regimen therapy VRd (BOR + lenalidomide + dexamethasone) but were less sensitive than parental cells (Figs. S7 E-F), suggesting they are still responsive to lenalidomide and dexamethasone. Co-administration of NOTCH3-ab or BT-GSI further enhanced VRd’s anti-MM efficacy in bones with parental or BOR-R cells but not in those bearing BOR-R MM cells (Figs. S7E-F). These findings suggest that, while NOTCH3 or pan-Notch inhibition does not restore sensitivity to BOR, it is an effective therapy for eliminating MM cells with intrinsic BOR resistance.

### Notch inhibitors simultaneously eliminate bortezomib-resistant and dormant MM cells

**Fig. 7:**
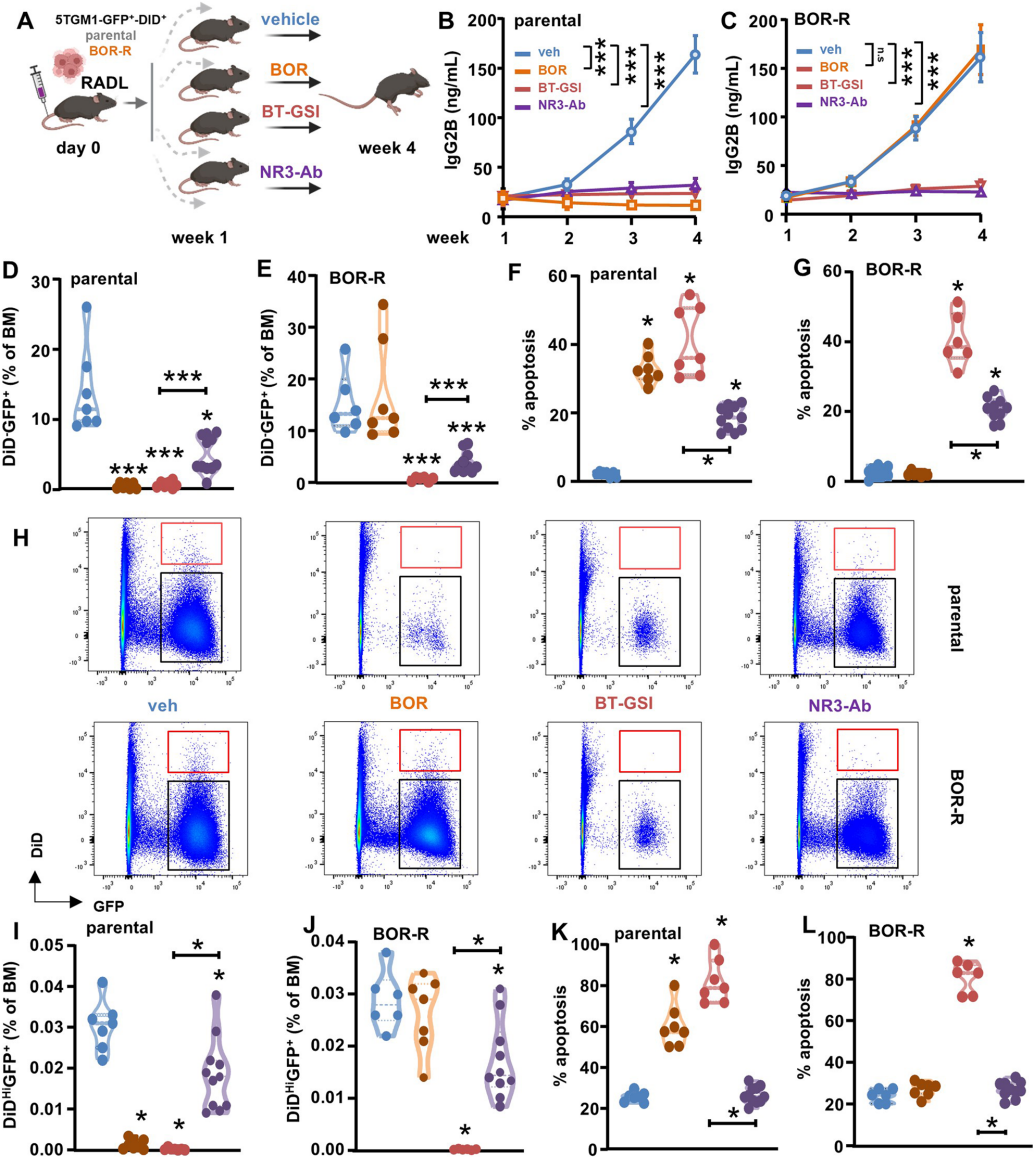
Notch inhibitors simultaneously eliminate dormant and BOR-resistant MM cells. **A** Experimental design. **B-C** Tumor progression, (**D-E**) prevalence and (**F-G**) percent of apoptotic proliferating (GFP+DiD-) parental and bortezomib resistant (BOR-R) 5TGM1 MM cells in the bone marrow, (**H**) representative flow cytometry plots of DiD and GFP channels, (**I-J**) prevalence and (**K-L**) percent of apoptotic dormant (GFP+DiDHi) parental and bortezomib resistant (BOR-R) 5TGM1 MM cells in the bone marrow from mice treated with vehicle, BOR, BT-GSI, or Notch3-ab (NR3-ab). Mice per group in parental 5TGM1 cohort: vehicle [], BOR [7], BT-GSI [7], NR3-ab [11]; in BOR-R cohorts: vehicle [6], BOR [7], BT-GSI [6], NR3-ab [10]. *p<0.05; **p<0.01; ***p<0.001 vs. vehicle by Lognormal One-way ANOVA followed by a Dunnett post hoc test (D-E) or ordinary One-Way ANOVA followed by a Tukey post hoc test (F-G, and I-L). Red rectangles indicate the GFP+DiDHi dormant population, and black rectangles represent the proliferating GFP+-DiD- population. n.s.=non-significant

To evaluate whether Notch inhibitors can simultaneously eliminate drug-resistant and dormant MM cells, we injected double-labeled GFP^+^DiD^+^ 5TGM1 parental or BOR-R cells intravenously into immunocompetent mice, creating a model in which dormant and BOR-resistant populations coexist. After 1 week, when tumors had engrafted, mice were randomized to treatment groups receiving vehicle, BOR, NOTCH3-ab, or BT-GSI for an additional 3 weeks (Fig. 7A). Mice injected with parental cells receiving vehicle and mice injected with BOR-R cells and treated with vehicle or BOR had a similar progression of tumor from 1 to 4 weeks (Figs. 7B-C). Only mice injected with parental cells showed a reduction in tumor burden in response to BOR. Mice injected with parental or BOR-R cells and treated with NOTCH3-ab or BT-GSI had an equivalent decrease in tumor compared to the vehicle group (Figs. 7B-C). Similar results were seen when quantifying the percentage of GFP^+^ proliferating cells in the bone marrow after 3 weeks of therapy (Figs. 7D-E). We observed increased apoptosis in proliferating cells with NOTCH3-ab or BT-GSI treatments, both in parental and BOR-R tumor-bearing mice. A similar increase in apoptosis was also noted in mice injected with parental cells and treated with BOR (Figs. 7F-G). BOR therapy reduced the percentage of dormant cells by 95% and induced apoptosis of dormant cells only in mice bearing parental cells. NOTCH3-ab and BT-GSI reduced the percentage of dormant cells by 40% and 99%, respectively, in both mice bearing parental or BOR-R cells, with BT-GSI having a more potent effect. However, only BT-GSI induced apoptosis in dormant cells (Figs. 7H-L). Together, these data demonstrate that inhibiting Notch signaling with NOTCH3-ab or BT-GSI reduces both the number of dormant and drug-resistant MM cells.

### Notch inhibitors protect from MM-induced bone disease

**Fig. 8:**
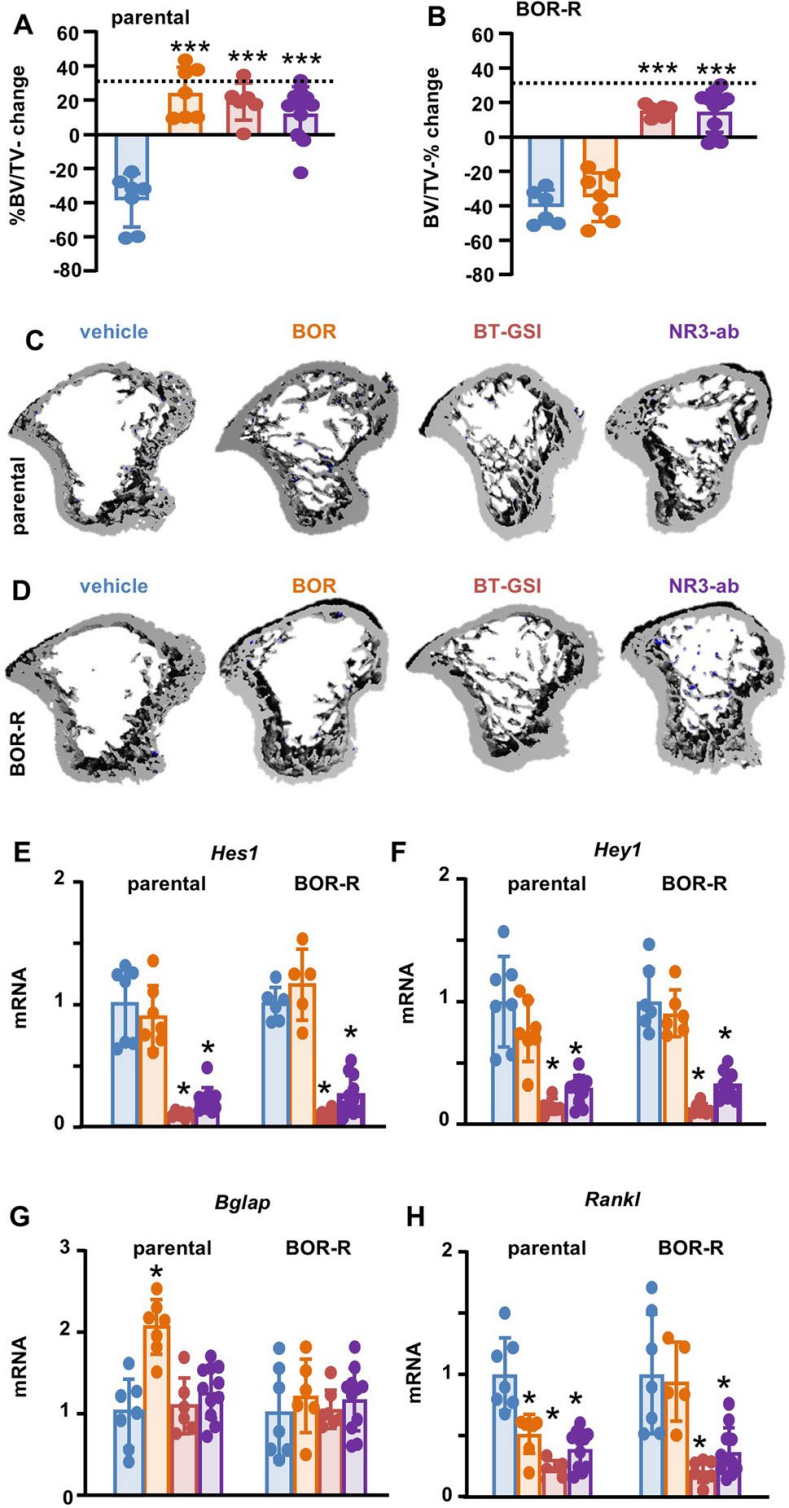
Notch inhibitors protect the skeleton from MM-induced bone disease. **A-B** Percent change in tibial cancellous bone mass from baseline (week 0) to endpoint (week 4) and (**C-D**) representative microCT 3D reconstruction images of the tibia from mice injected with 5TGM1 parental or BOR-R cells and treated with vehicle, BOR, BT-GSI, or Notch3-ab. Mice per group in parental 5TGM1 cohort: vehicle [7], BOR [7], BT-GSI [6], NR3-ab [11]; in BOR-R cohorts: vehicle [6], BOR [7], BT-GSI [7], NR3-ab [11]. mRNA gene expression of (**E**) *Hes1*, (**F**) *Hey1*, (**G**) *Bglap*, and (**H**) *Rankl* in the L4 vertebrae of mice injected with 5TGM1 parental or BOR-R cells and treated with vehicle, BOR, BT-GSI, or Notch3-ab. Mice per group in parental 5TGM1 cohort: vehicle [7], BOR [7], BT-GSI [6], NR3-ab [11]; in BOR-R cohorts: vehicle ( [6]– [7]), BOR ( [5]– [6]), BT-GSI [7], NR3-ab [11]. Samples with poor RNA quality and outlier data points were excluded from the gene expression analysis. *p < 0.05; **p < 0.01; ***p < 0.001 vs. veh by ordinary one-way ANOVA followed by a Tukey post hoc test

Because bone destruction is a hallmark of MM and can worsen during disease relapse, we examined the impact of Notch inhibitors on bone health. Mice injected with parental cells and treated with vehicle, and mice injected with BOR-R cells and treated with vehicle or BOR had a ~ 40% reduction in bone mass. BOR only prevented bone loss in mice injected with parental cells. Mice treated with BT-GSI or NOTCH3-ab, regardless of whether they were injected with parental or BOR-R cells, did not lose bone and exhibited reduced expression of *Rankl* (Figs. 8A-D, H). In addition, NOTCH3-ab and BT-GSI reduced Notch activation similarly in bone from mice injected with parental or BOR-R cells (Figs. 8E-F). Moreover, BOR therapy increased *Bglap*, a marker of osteoblasts and bone formation, only in mice bearing parental MM cells (Fig. 8G). This data demonstrates that NOTCH3-ab and BT-GSI can effectively reduce tumors, eliminate dormant and therapy-resistant cells, and protect the skeleton.

## Discussion

Drug resistance is the leading cause of relapsed/refractory disease and decreased survival, and is a major obstacle to more successful clinical outcomes in MM. Moreover, the clinical observation that dormant cells can drive relapse [9] indicates that curative therapies must overcome not only tumor resistance but also dormancy. In this study, through a combination of preclinical models and clinical samples, we identify Notch signaling as a shared vulnerability in BOR-resistant and dormant MM cells. Collectively, these findings provide a strong rationale for developing strategies based on safe Notch inhibitors to prevent disease relapse and extend remission in MM patients.

The detection of dormant clones with relapse capacity in MM patients [9], together with the prognostic impact of the dormancy gene signature identified in our study, is consistent with the notion that dormant cells have a significant role in disease relapse. However, eradicating dormant cells is challenging, as these cells are largely unresponsive to conventional cancer therapies such as melphalan [8], which primarily target actively dividing cells. Prior work identified myeloid genes and Axl as therapeutic targets for dormant cells [7]. Our study demonstrates that dormant MM cells also exhibit upregulation of Notch components and that Notch inhibition with BT-GSI effectively eliminates these dormant cells, overcoming niche-induced dormancy. Notch has been previously implicated in regulating the escape of breast cancer cells from dormancy [33]. In the context of MM, our findings demonstrate that the Notch pathway is crucial for the survival of dormant MM cells, as evidenced by the robust induction of apoptosis triggered by BT-GSI. NOTCH3-ab also reduced the number of dormant cells, although to a lesser extent than BT-GSI, and through a mechanism that appears independent of apoptosis. One possible explanation for the distinct effects of BT-GSI and NOTCH3-ab is that different Notch receptors may play unique roles in dormant cells, as we have previously observed in actively proliferating MM cells [10, 25]. Our data suggest that NOTCH3 may play a more significant role in maintaining cell cycle arrest in dormant cells, and that inhibiting NOTCH3 could potentially awaken these cells from their dormant state. Other Notch receptors (NOTCH1, NOTCH2, or NOTCH4) targeted by BT-GSI but not by NOTCH3-ab may be more relevant for integrating pro-survival signals that keep dormant cells alive. Future studies are warranted to explore these possibilities and to elucidate the specific role of NOTCH3 signals in the initiation, maintenance, and reactivation of MM cell dormancy programs.

BOR has a demonstrated survival benefit in MM patients. However, the overall response rate to BOR trials is lower than 50%, suggesting that a subset of patients exhibits intrinsic resistance to BOR or develops resistance during the course of the disease [34, 35]. Thus, identifying synergistic drug combinations or alternative vulnerabilities is needed to overcome BOR resistance. In this study, we observed a negative correlation between the expression levels of γ-secretase complex components at diagnosis, which activate Notch receptors, and clinical responses to BOR-based therapies. We also found that MM cells with intrinsic drug resistance to BOR have upregulation of several Notch components, including receptors and γ-secretase complex subunits. These findings demonstrate the dysregulation of Notch signaling in BOR-resistant MM cells. Importantly, Notch inhibition with BT-GSI or NOTCH3-ab eliminated BOR-resistant MM cells, although it did not restore BOR sensitivity in BOR-R cells, and induced apoptosis in primary CD138^+^ cells isolated from MM patients with partial responses to therapy. These results identify the Notch pathway as a targetable vulnerability in BOR-resistant cells and suggest that Notch inhibition may offer an alternative therapeutic strategy for patients with BOR-refractory disease.

An interesting aspect of this work is the emergent role of NOTCH3 in MM therapeutic resistance. We previously reported that NOTCH3 integrates pro-survival signals from the tumor niche, conferring secondary resistance to BOR to MM cells [10]. We also showed that high *NOTCH3* expression at diagnosis is associated with poor responses to BOR-based therapies [10]. In the current study, we demonstrate that *NOTCH3* is preferentially upregulated in MM cells with intrinsic BOR resistance, and that treatment with NOTCH3-ab induces apoptosis in actively proliferating BOR-R MM cells. Notably, NOTCH3-ab produced comparable reductions in tumor burden compared to BT-GSI, a pan-Notch inhibitor that targets all Notch receptors. However, we also observed upregulation of other Notch receptors in BOR-R cells, which may contribute to intrinsic resistance and warrant further investigation.

The clonal and spatial heterogeneity of MM poses a challenge to treatment, as therapies effective for one subclone may fail against others, resulting in treatment resistance and relapse. Previous research, including our own, has shown that Notch signals contribute to secondary resistance mediated by cells of the tumor niche, with Notch inhibition restoring sensitivity to therapy [10–15]. The current study is among the first to show in vivo that dormant and intrinsically drug-resistant MM cells are also sensitive to Notch inhibition. We demonstrate that the shared dependence of MM cells on Notch signals for survival enables Notch inhibitors, such as BT-GSI and NOTCH3-ab, to simultaneously eliminate various drug-resistant populations, regardless of whether resistance arises from intrinsic mechanisms, extrinsic factors, or dormancy programs. Moreover, these Notch inhibitors also offer bone protective effects, which could further improve the survival and quality of life of patients. Importantly, these Notch inhibitors do not exhibit the typical toxicities that have hindered the clinical approval of Notch inhibitors [36, 37]. Our previous studies have demonstrated that treatment with BT-GSI does not cause significant side-effects, including changes in body weight, complete blood cell counts, gastrointestinal toxicity, or serum markers of toxicity [18, 26]. Additionally, administration of NOTCH3-ab does not produce the significant toxicities commonly associated with broader Notch inhibition [38, 39]. Taken together, our findings suggest that BT-GSI and NOTCH3-ab may represent promising, safer strategies to inhibit Notch signaling in the MM niche.

## Conclusions

In conclusion, our study identifies the Notch signaling pathway as a shared vulnerability across various types of drug-resistant cells and demonstrates its potential to eliminate coexisting dormant and therapy-resistant MM populations. Our findings suggest that treatment with NOTCH3-ab or BT-GSI may circumvent clonal drug resistance heterogeneity, a challenge often encountered with current regimes. These results provide a compelling rationale for developing safe Notch inhibitors as a new therapeutic tool to expand the arsenal of treatment options and improve clinical outcomes in relapsed/refractory patients.

## Supplementary Information

Supplementary Material 1.

## Data Availability

The IA18 datasets used for the analyses described in this work were downloaded from the Multiple Myeloma Research Foundation CoMMpass study ( [www.themmrf.org](http:/www.themmrf.org) ) researcher gateway. The MRD and MM cell dormancy scRNAseq datasets analyzed in this study were obtained from GEO accession GSE70399 and Bioproject PRJNA453652 (7), respectively. All other raw data are available from the corresponding author on reasonable request.

## Abbreviations

MM: Multiple myeloma
MRD: Minimal Residual Disease
GSI: Gamma secretase inhibitor
BT-GSI: Bone-targeted Notch inhibitor
Scl-ab: Neutralizing antibody against sclerostin
BOR: Bortezomib
BOR-R: Bortezomib-resistant
VRd: Triple regimen bortezomib + lenalidomide + dexamethasone
NOTCH3-ab: NOTCH3 neutralizing antibody
