# Supplementary material for: Targeting NOTCH3 to eradicate dormant and therapy-resistant multiple myeloma cells
Source: J Exp Clin Cancer Res. 2026 Jan 5;45:31. doi: 10.1186/s13046-025-03630-1 (PMC12870532; doi:10.1186/s13046-025-03630-1)
Supplement: Supplementary file 1 — Supplementary Material 1. [file 13046_2025_3630_MOESM1_ESM.docx]

Supplementary Materials for

**Targeting NOTCH3 to eradicate dormant and therapy-resistant multiple myeloma cells**

Hayley M. Sabol, Bethany C. Paxton, Aric Anloague, Japneet Kaur, Mattie R. Nester, Sharmin Khan, James Smith, Peter I. Croucher, Michelle M. McDonald, Corey Montgomery, Jeffrey B. Stambough, C. Lowry Barnes, Elena Ambrogini, Frank H. Ebetino, Carolina Schinke, Cody Ashby, and Jes**us** Delgado-Calle

*Corresponding author email: Jdelgadocalle@uams.edu

**The PDF file includes:**

Figure S1. Pharmacokinetics and pharmacodynamics of BT-GSI.

Figure S2. High dormancy and proliferation signatures are associated with poor survival.

Figure S3. Characterization of the immunodeficient and immunocompetent mouse models of MM cell dormancy.

Figure S4. Bone-targeted Notch inhibition decreases proliferating MM cells.

Figure S5. Scl-ab does not prevent BT-GSI anti-MM effects on proliferating and dormant MM cells or affect the rate of MM cells entering dormancy as a single agent. .

Figure S6. Bortezomib-resistant cells exhibit upregulation of Notch components and are sensitive to Notch inhibition.

Figure S7. Notch inhibitors reduced tumor burden in bones bearing Bortezomib-resistant cells cultured ex vivo.

Table S1. Gene lists for dormancy and proliferation signatures.

Supplementary file. Uncropped western blots for Figure 5C.

**Fig. S1. Pharmacokinetics and pharmacodynamics of BT-GSI.** The concentration of free GSI from the serum and bone lysate from mice treated with gamma-secretase inhibitor (GSI) or the bone-targeted Notch inhibitor (BT-GSI) for (**A**) 4 weeks (n=7 mice/group) and (**B**) after a single dose of BT-GSI (n=6-7 mice/group). (**C**) Gene expression of Notch target genes (*Hey1*, *Hey2*, *Hes5*) and concentration of free GSI were analyzed up to 24 hours after a single dose of BT-GSI. *p< 0.05; **p< 0.01; ***p<0.001 vs. GSI by ordinary t-test.

**Figure S2. High dormancy and proliferation signatures are associated with poor survival.** (**A**) Kaplan-Meier plot of overall survival (OS) and progression-free survival (PFS) in newly diagnosed patients with high and low proliferation gene signatures. (**B**) Overall and progression-free survival of newly diagnosed patients with a high dormancy and high proliferation gene signature (red line), high proliferation and low dormancy gene signature (green line), low proliferation and high dormancy gene signature (teal line), and low proliferation and low dormancy gene signature (purple line). Data were analyzed using a Log-rank (Mantel-Cox) test.

**Figure S3. Characterization of the immunodeficient and immunocompetent mouse models of MM cell dormancy.** (**A**) Gating for detecting proliferating and dormant populations in flow cytometry samples. (**B-C**) Percentage of GFP^+^DiD^-^ proliferating and dormant GFP^+^DiD^Hi^ MM cells in the bone marrow 3 and 4 weeks after 5TGM1 cell inoculation. Red rectangles indicate the GFP^+^DiD^Hi^ MM dormant population, and black rectangles represent the proliferating GFP^+^-DiD^-^ MM population. (**D**) Cell cycle analysis in GFP^+^DiD^-^ proliferating and GFP^+^DiD^Hi^ dormant MM cells in the bone marrow and (**E**) percentage of Axl^+^ cells in dormant and proliferating MM cells at week 3. n=3-6 mice/group. *p< 0.05; **p< 0.01; ***p<0.001 vs. vehicle by Lognormal Welch’s T-test (B) or ordinary T-test (C, E).


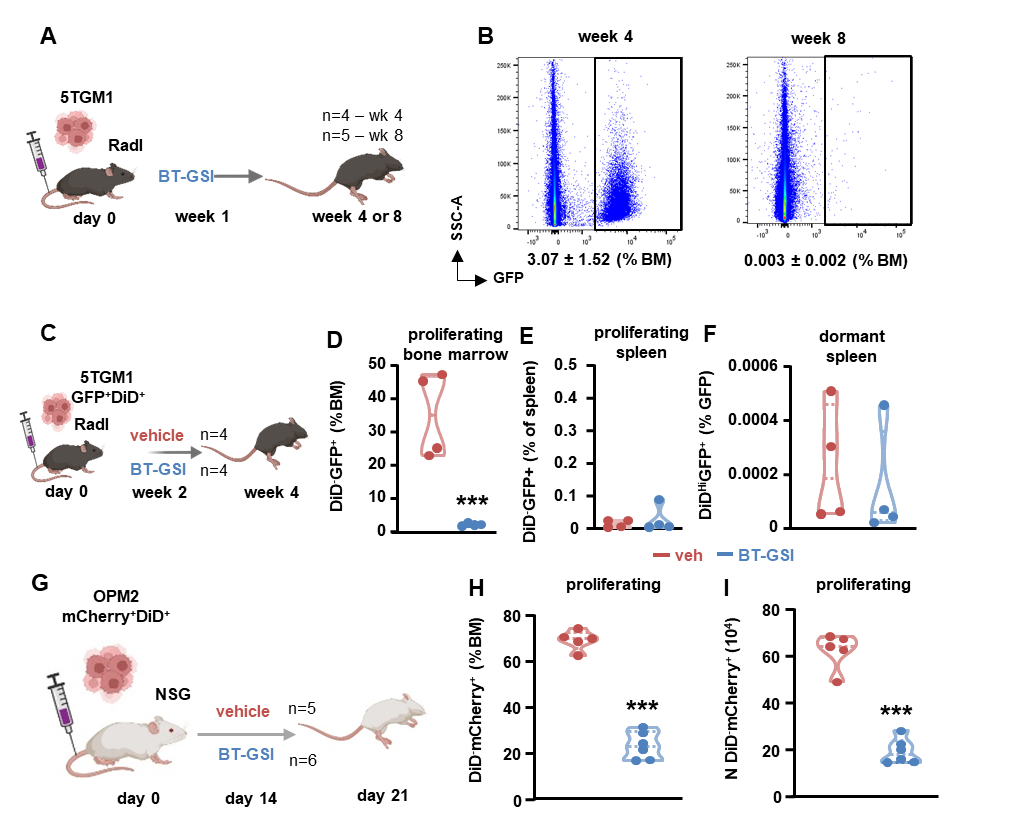


**Figure S4. Bone-targeted Notch inhibition decreases proliferating MM cells.** (**A**) Immunocompetent mouse model experimental design. (**B**) Quantification and representative flow cytometry plots of GFP^+^ 5TGM1 MM cells in the bone marrow of mice receiving BT-GSI for 4 (n=4) or 8 (n=5) weeks. (**C**) Immunocompetent mouse model. (**D-F**) Percentage of proliferating GFP^+^DiD^-^ cells in bone marrow, and percentage of proliferating GFP^+^DiD^-^ and dormant GFP^+^DiD^Hi^ MM cells in the spleen after 2 weeks. n=4 mice/group. (**G**) Immunodeficient mouse model experimental design. (**H-I**) Percentage and number of mCherry^+^DiD^-^ human OPM2 MM cells in the bone marrow from mice receiving vehicle (veh) or bone-targeted Notch inhibitor (BT-GSI) for 1 week. n=5-6 mice/group. *p< 0.05; **p< 0.01; ***p<0.001 vs. vehicle by Lognormal Welch’s T-test (D, H) or ordinary T-test (E, F, I).


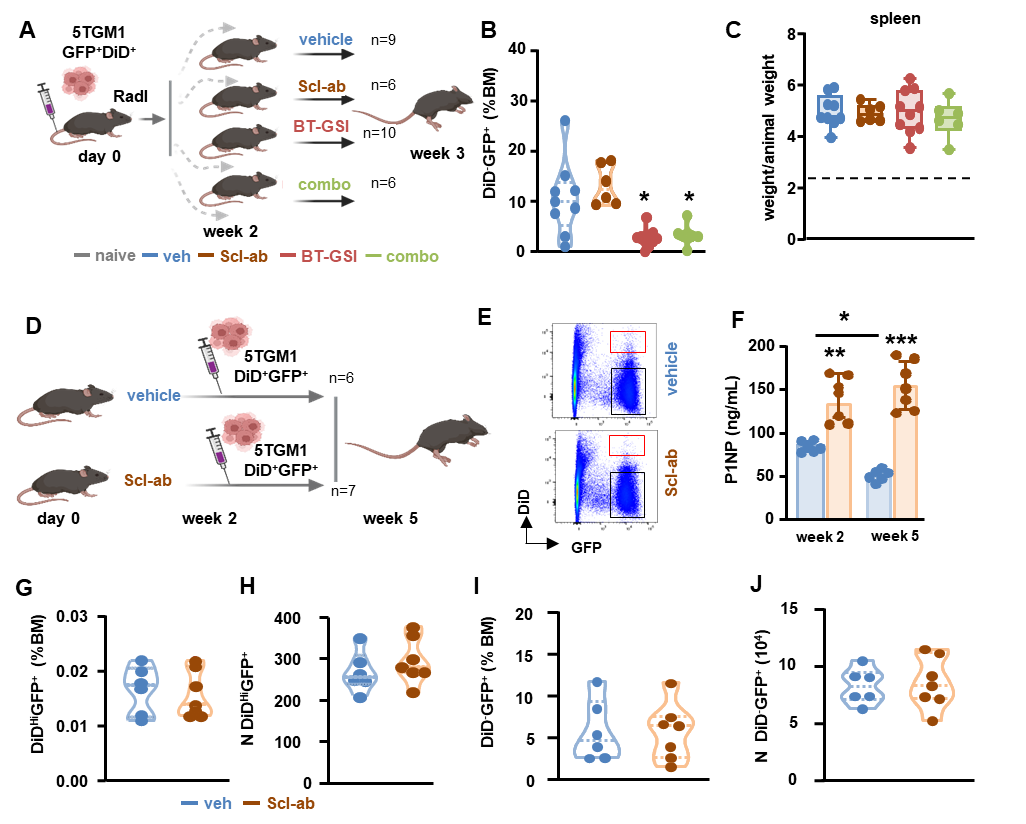


**Figure S5. Scl-ab does not prevent BT-GSI anti-MM effects on proliferating and dormant MM cells or affect the rate of MM cells entering dormancy as a single agent.** (**A**) Immunocompetent mouse model experimental design. (**B**) Percentage of GFP^+^DiD^-^ proliferating 5TGM1 MM cells in the bone marrow and (**C**) weight of the spleen in mice receiving vehicle (veh), anti-sclerostin antibody (Scl-ab), bone-targeted Notch inhibitor (BT-GSI), or a combination of BT-GSI and Scl-ab (combo) for 1 week. The dashed line indicates the average spleen weight of naïve mice. n=6-10 mice/group. (**D**) Immunocompetent mouse model experimental design. (**E**) Representative flow cytometry plots. Red rectangles indicate the GFP^+^DiD^Hi^ MM dormant population, and black rectangles represent the proliferating GFP^+^DiD^-^ MM population. (**F**) Serum levels of the bone formation marker P1NP, and (**G-H**) percentage and number of dormant GFP^+^DiD^Hi^ 5TGM1 MM cells and proliferating GFP^+^DiD^-^ 5TGM1 MM cells in the bone marrow of mice treated with veh or Scl-ab for 5 weeks. n=6-7 mice/group. *p<0.05; **p<0.01; ***p<0.001 vs. vehicle by Lognormal One-way ANOVA followed by a Dunnett post hoc test (B), ordinary One-Way ANOVA followed by a Tukey post hoc test (C, D), Lognormal Welch’s T-test (I) or ordinary T-test (G,H,J).

**Figure S6. Bortezomib-resistant cells exhibit upregulation of Notch components and are sensitive to Notch inhibition.** (**A**) mRNA gene expression of Notch receptors and target genes in 5TGM1 parental and bortezomib-resistant (BOR-R) cells. n=4/group. (**B-E**) mRNA gene expression of Notch receptors, target genes, and γ-secretase complex components in RPMI-8226 and U266 parental and BOR-R cells. n=4/group. (**F-G**) Percentage of dead cells in parental and BOR-R RPMI-8226 and U266 MM cells treated with veh, BOR, GSI, Notch3-ab (NR3-ab), BOR+GSI, or BOR+NR3-ab for 48h. n=4/group. *p<0.05; **p<0.01; ***p<0.001 vs. parental by ordinary t-test (A-E) and ordinary two-way ANOVA (F-G), followed by a Tukey post hoc test.

**
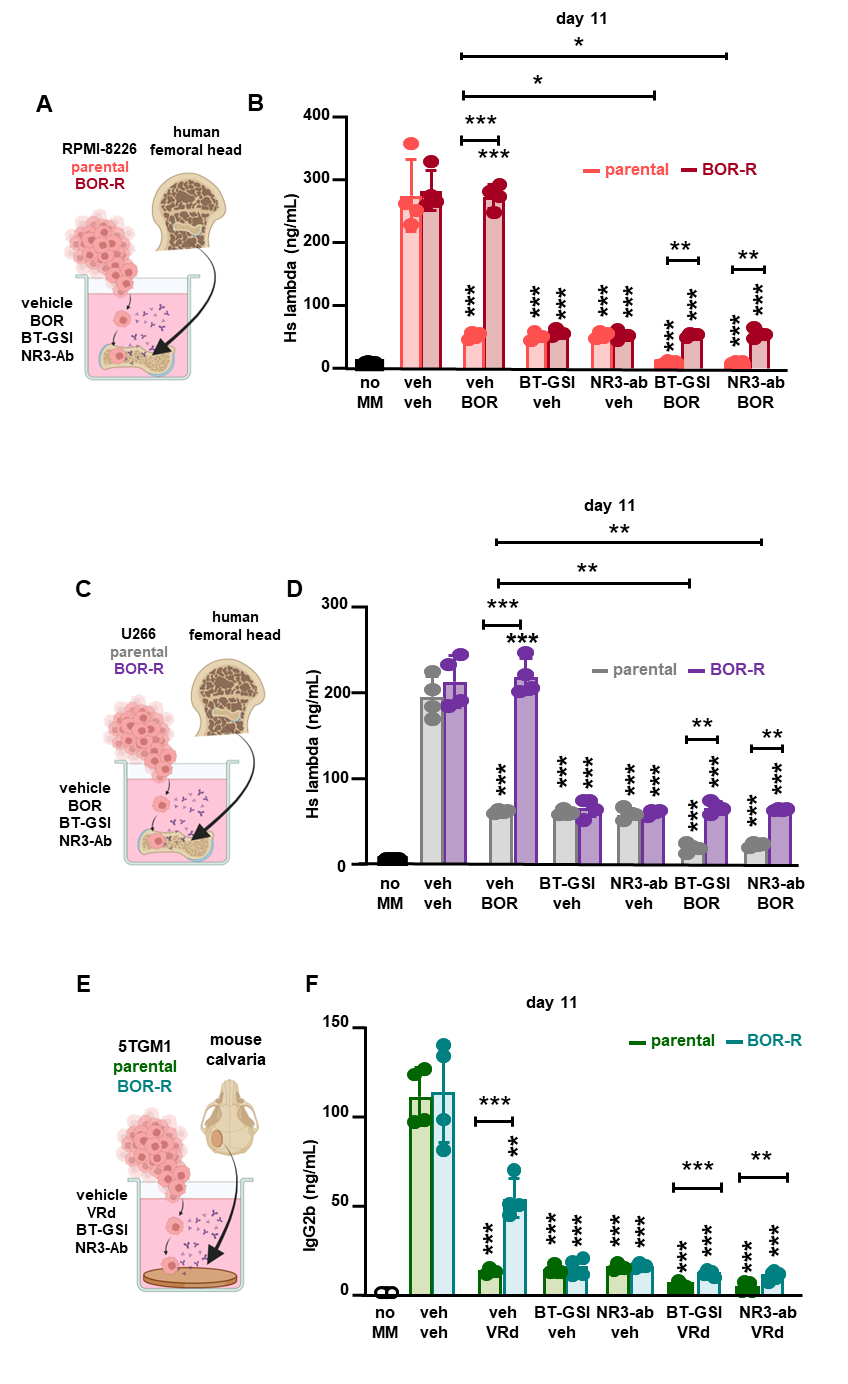
**

**Figure S7. Notch inhibitors reduced tumor burden in bones bearing Bortezomib-resistant cells cultured *ex vivo*.** Ex vivo experimental designs (**A, C**) and levels of the tumor biomarker lambda chain in the conditioned media of *ex vivo* human bone samples infiltrated by human (**B**) RPMI-8226 or (**D**) U266 parental or bortezomib resistant (BOR-R) cells and treated with vehicle, BOR, BT-GSI, Notch3-ab (NR3-ab), BOR+BT-GSI, or BOR+NR3-ab after 11 days of culture. (**E-F**) Experimental design and levels of the tumor biomarker IgG2b in *ex vivo* murine calvarial bones infiltrated by 5TGM1 parental or BOR-R cells and treated with vehicle, VRd, BT-GSI, NR3-ab, VRd+BT-GSI, or VRd+NR3-ab after 11 days of culture. n=4-5/group. *p<0.05; **p<0.01; ***p<0.001 vs. parental (veh) by ordinary two-way ANOVA, followed by a Tukey post hoc test.

**Table S1. Gene lists for dormancy and proliferation signatures.**

| **Gene signatures** | | |
| --- | --- | --- |
| **Proliferation** | **Dormancy -down** | **Dormancy-up** |
| *TOP2A* | *TSPEAR* | *RNF152* |
| *BIRC5* | *DOCK5* | *USP25* |
| *CCNB2* | *UPRT* | *ERG* |
| *NEK2* | *MYLIP* | *MAGEE1* |
| *ANAPC7* | *METTL22* | *AMER1* |
| *STK6* | *HSPH1* | *PSMA8* |
| *BUB1* | *ELOVL4* | *SVIP* |
| *CDC2* | *MVB12B* | *PPM1H* |
| *C10orf3* | *SUSD2* | *ABCC3* |
| *ASPM* | *MON1B* | *TNFAIP2* |
| *CDCA1* | *PTPRU* | *RRAGB* |
|  | *GCH1* | *IGSF6* |
|  | *IL15* | *HCK* |
|  | *CDK20* | *CD19* |
|  | *RNF144B* | *SLC25A4* |
|  | *HS6ST1* | *HPGD* |
|  | *HIC2* | *PDPR* |
|  | *GALNT13* | *KLHL6* |
|  | *NUDT6* | *SLC43A2* |
|  | *MRGPRE* | *BATF* |
|  | *OLFML3* | *SERPINB10* |
|  | *PROM1* | *ZCCHC2* |
|  | *TMEM62* | *PLA2G15* |
|  | *TMEM236* | *ANXA2* |
|  | *IGFBP7* | *VPREB3* |
|  | *LCMT2* | *CD5L* |
|  | *GEMIN8* | *IFIT2* |
|  | *SCO1* | *CD2* |
|  | *EARS2* | *CTSG* |
|  | *ADCK2* | *TGFB1I1* |
|  | *GPAM* | *RCSD1* |
|  | *SLC17A7* | *AIF1* |
|  | *SELP* | *AXL* |
|  | *E2F6* | *GGH* |
|  | *NOL4L* | *CPM* |
|  | *MKS1* | *PMP22* |
|  | *SARM1* | *MEGF9* |
|  | *ATRN* | *PTGR1* |
|  | *SAMD10* | *GNG10* |
|  | *NUMBL* | *SLC9A5* |
|  | *CASP7* | *MYBL1* |
|  | *ARL5C* | *ARMCX5* |
|  | *ZNF438* | *PTPRCAP* |
|  | *GSTK1* | *CD4* |
|  | *VDR* | *GLUL* |
|  | *SPEF2* | *SLC44A2* |
|  | *TMC4* | *C19orf38* |
|  | *POPDC2* | *HADH* |
|  | *TMEM53* | *CCDC170* |
|  | *PIH1D2* | *HLA-DOA* |
|  | *NUGGC* | *SLC12A1* |
|  | *WARS2* | *GAS7* |
|  | *NOXRED1* | *TMEM119* |
|  | *C7orf25* | *LRPAP1* |
|  | *SLC25A21* | *LY86* |
|  | *BACE1* | *PRKCB* |
|  | *CRELD1* | *EMB* |
|  | *MAP1A* | *APOC2* |
|  | *DENND2D* | *SMIM5* |
|  | *ANO1* | *STARD4* |
|  | *GNAT2* | *SPIB* |
|  | *PLEKHN1* | *GPR65* |
|  | *SH2D4B* | *PPARG* |
|  | *PLD1* | *EVL* |
|  | *CTNS* | *ADAP2* |
|  | *GABRR2* | *ITPR1* |
|  | *COMP* | *SAMD9L* |
|  | *TEKT2* | *TMED3* |
|  | *ANKRD49* | *FGR* |
|  | *CCDC90B* | *ACP5* |
|  | *TIGD3* | *P2RY13* |
|  | *TMEM150A* | *IFI44* |
|  | *TNNT1* | *TRAF5* |
|  | *POC1B* | *LCP2* |
|  | *MYO15B* | *NUCB2* |
|  | *BICD1* | *GLIPR1* |
|  | *AMACR* | *HEBP1* |
|  | *EOGT* | *TIFA* |
|  | *OVGP1* | *MYL4* |
|  | *CAMKMT* | *FCGR1A* |
|  | *RMDN2* | *S100A10* |
|  | *SLC27A3* | *MYH14* |
|  | *ASAP3* | *RAG1* |
|  |  | *CYB5RL* |
|  |  | *PCDHGA5* |
|  |  | *CLEC4A* |
|  |  | *HFE* |
|  |  | *S100A6* |
|  |  | *S100A4* |
|  |  | *TIFAB* |
|  |  | *IL18BP* |
|  |  | *NR1H3* |
|  |  | *GAPT* |
|  |  | *IFIH1* |
|  |  | *PHF13* |
|  |  | *C1QA* |
|  |  | *EBF1* |
|  |  | *CD84* |
|  |  | *BIN1* |
|  |  | *RGS2* |
|  |  | *PER2* |
|  |  | *SLC11A1* |
|  |  | *SDC3* |
|  |  | *DUSP10* |

**Supplementary File. Uncropped and unedited western blots for Figure 5C.**
